# Mortality before and during COVID-19 outbreak in the Federation of Bosnia and Herzegovina: Šeila Lagarija

**DOI:** 10.1093/eurpub/ckac131.573

**Published:** 2022-10-25

**Authors:** Š Cilović-Lagarija, S Musa, S Skočibušić

**Affiliations:** Institute of Public Health for FBiH, Sarajevo, Bosnia and Herzegovina

## Abstract

**Background:**

Mortality data are essential for monitoring population health and is one of the most important data for evaluation and comparison of health status at the local, national, and international level.

Objective: We analysed all-cause mortality data in the Federation of Bosnia and Herzegovina (FBiH) for the period 2016-2021 and compared it with mortality occurred during the COVID-19 pandemic, in 2020 and 2021.

**Methods:**

Using data on all-cause deaths for the period 2016-2021, obtained from the Institute for Statistics of the Federation of Bosnia and Herzegovina, we compared annual number of deaths (all-ages) and death rates during the 2020 and 2021 to pre-pandemic years.

**Results:**

In 2016 the reported number of death was 21,146, in 2017 was 21,942, in 2018 was 21,691, and in 2019 was 22,024, and during the pandemic period in 2020 and 2021, 26,026 and 29,086 deaths were reported respectively. In 2020, 4,115 more deaths has been reported (15,8%), and in 2021 more 6,438 death (22,1%) compared with period 2016-2019. In FBiH in 2021, the death rate per 100,000 inhabitants was 1,341.2 and it is recorded an increase compared to 2020 when it had a value of 1,208.3 while in 2016 the value was 951.7.

**Conclusions:**

A large proportion of increased mortality during pandemic was probably caused directly by COVID-19. However, the pandemic also resulted in deaths that would otherwise not have occurred (indirect deaths) due lack of access to medical services when hospitals were overwhelmed and changes in health seeking behaviour. An in-depth investigation of the underlying causes of the high excess mortality should be conducted to inform changes in the health care system and efforts to prevent severe COVID-19 through vaccination of vulnerable groups should be a priority.

*This abstract is support by ‘BoCO-19 - The Burden of Disease due to COVID-19'. Project is coordinated/led by Robert Koch Institute and supported by the WHO Regional Office for Europe.

**Key messages:**

• During the two years of the COVID-19 pandemic, population in FBiH had a significant increase in all-cause mortality.

• The direct standardized death rate for all causes and age groups per 100,000 inhabitants in 2020 for FBiH was 818.0 and it is slightly higher compared to the EU average.

